# What is pathogen-mediated insect superabundance?

**DOI:** 10.1098/rsif.2020.0229

**Published:** 2020-09-09

**Authors:** Ruairí Donnelly, Christopher A. Gilligan

**Affiliations:** Department of Plant Sciences, University of Cambridge, Cambridge CB2 3EA, UK

**Keywords:** plant pathogen, manipulation, epidemiology, vector, phytophagous, superabundance

## Abstract

When increasing abundance of insect vectors is manifest across multiple fields of a crop at the landscape scale, the phenomenon is sometimes referred to as insect superabundance. The phenomenon may reflect environmental factors (i.e. *environmentally mediated insect superabundance*, EMiS), including climatic change. A number of pathogens, however, are also known to modify the quality of infected plants as a resource for their insect vectors. In this paper, we term increasing vector abundance when associated with pathogen modification of plants as *pathogen-mediated insect superabundance* (henceforth PMiS). We investigate PMiS using a new epidemiological framework. We formalize a definition of PMiS and indicate the epidemiological mechanism by which it is most likely to arise. This study is motivated by the occurrence of a particularly destructive cassava virus epidemic that has been associated with superabundant whitefly populations in sub-Saharan Africa. Our results have implications for how PMiS can be distinguished from EMiS in field data. Above all, they represent a timely foundation for further investigations into the association between insect superabundance and plant pathogens.

## 1. Introduction

There is empirical evidence for increasing abundance of whitefly over large areas of Africa [1–4]. This has several important consequences for crop production. High densities of the insect can cause damage to plants through their feeding activity, and, in addition, whitefly are vectors of important viral pathogens of major subsistence crops, such as cassava. When increasing abundance of vectors is manifest across multiple fields of a crop at landscape scales, the phenomenon is sometimes known as insect superabundance [1,2,5,6]. The increase in abundance may be associated with a range of factors, including climatic change [3] (termed here as *environmentally mediated insect superabundance*, EMiS). But there is evidence that pathogen infection of plants can itself increase the abundance of vectors on infected plants [7,8]. In this report, we examine the epidemiological dynamics of pathogens that modify plants as a resource for vectors. Based on epidemic dynamics, our goal is to identify the epidemiological mechanism that is most favourable for the occurrence of *pathogen-mediated insect superabundance* (i.e. PMiS, as distinct from EMiS). We motivate the problem using whitefly-borne begomoviruses (which include, for instance, cassava mosaic virus and tomato leaf curl virus), which are well studied experimentally, and in which regional epidemic spread has coincided with superabundance of the polyphagous tabacco whitefly *Bemisia tabaci*, the species complex that transmits these viruses [9].

When superabundance is mediated by a pathogen, the increased density of vectors on infected plants leads to more successful transmission of infection, as increased numbers of insect vectors disperse from infected to surrounding healthy plants. This, in turn, leads to a cycle of increased vector density, leading to more infected plants that give rise to more vectors. A range of epidemiological mechanisms have been proposed whereby pathogen infection could modify plants to support higher densities of vectors. The underlying biological mechanisms are usually investigated using molecular and physiological tools. Typically, these analyses are supported by experiments that demonstrate correlations between vector density and plant traits. For example, high insect densities have been linked to high amino-acid concentrations in virus-infected cassava phloem [10,11]; high insect densities have been linked to altered plant defence hormones in virus-infected tobacco and tomato plants [12,13]; and increased egg production has been found on virus-infected tomato plants [14]. Here we focus on the consequences of such pathogen-induced changes in plant traits (i.e. epidemiological mechanisms) on the population dynamics of vector and pathogen. In particular, we evaluate the ability of each epidemiological mechanism to induce elevated insect abundance at the landscape scale (i.e. PMiS).

We approach the problem of establishing the epidemiological mechanisms that lead to PMiS by first deriving the vector dynamics for a given incidence of the pathogen among plants (see Material and methods: Vector dynamics), then deriving the pathogen dynamics for a given abundance of the vector (see Material and methods: Epidemiological dynamics). Using the resulting set of equations, we provide a quantitative definition of PMiS and use it to differentiate the effects of the distinct epidemiological mechanisms. The proposed epidemiological mechanisms that alter vector dynamics encompass changes to multiplication rate, carrying capacity and preference of the insect vector for infected plants. With this approach, it is possible to distinguish the roles of vector and pathogen in accounting for superabundance and to evaluate methods for detecting PMiS in empirical survey data. We discuss the implications of our results for the unprecedented increase in abundance of the *B. tabaci* whitefly, the vector of multiple cassava viruses, that has occurred in East and Central Africa since the 1990s [1,5,9], where 100-fold increases in *B. tabaci* abundance together with the accompanying cassava mosaic disease (CMD) pandemic caused crops to be abandoned, leading to widespread food shortages and famine-related deaths [2,15].

## 2. Material and methods

### 2.1. Vector dynamics

**Figure 1.**
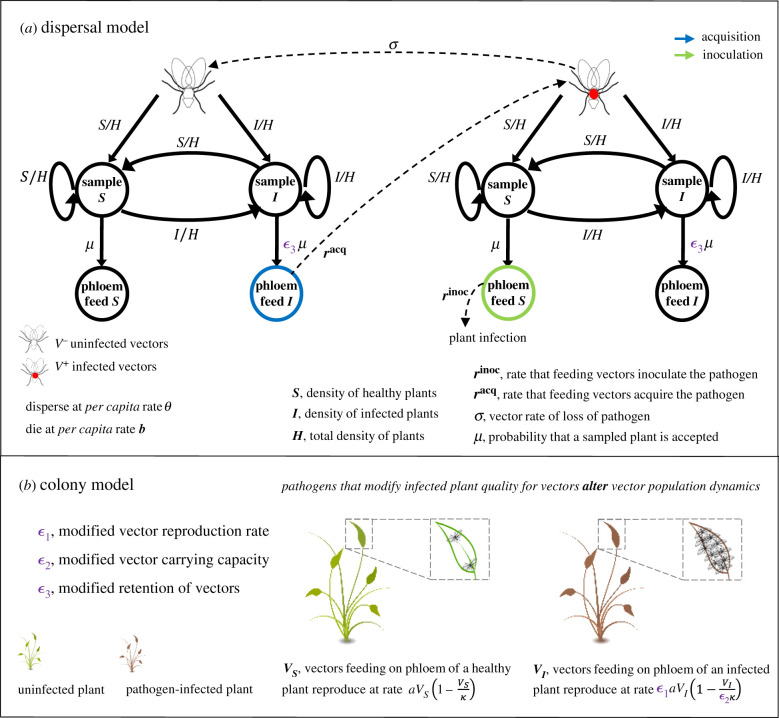
Pathogens that modify plants as a resource for vectors may influence the dispersal or reproductive processes (*a*,*b*). The pathosystem model, which combines (*a*) and (*b*), comprises (*a*) a Markov chain model of vector feeding dispersals (with associated pathogen transmission) and (*b*) vector reproduction when the insect vector is settled and feeding. Pathogen infection of plants determines vector abundance as a consequence of altered reproductive processes on infected plants (if *ε*_1_ ≠ 1 or if *ε*_2_ ≠ 1 in (*a*)) or as a consequence of altered retention of vectors after they have sampled infected plants (if *ε*_3_ ≠ 1 in (*b*)).

In order to investigate the ability of putative epidemiological mechanisms to lead to insect superabundance, we model the joint population dynamics of insect colonies and pathogen epidemics. For simplicity, the complex life stages of specific insect vectors are not incorporated here; instead, we focus on vector dynamics of the adult insects. Phytophagous insect vectors of plant pathogens, such as whitefly, aphids and thrips, move between host plants assessing their acceptability through probing behaviour. The insect vectors settle and feed on the phloem tissue of a plant’s vascular system if the plant is acceptable, and, when settled, reproduce (figure 1*a*,*b*). We consider a general case in which pathogen modification of plants affects the population dynamics of vectors, leading to relatively high vector abundance on infected compared with healthy plants (figure 1*b*). Fundamentally, the insect population dynamics involve reproduction, mortality and dispersal with density dependence constraining population growth of the vector at the level of individual plants [16]. The major limitation on phytophagous insect growth rates relates to the nutritional status of insect food. If PMiS occurs, leading to elevated insect abundance, it is therefore reasonable to assume that some aspect of growth or dispersal depends upon the infectious state of the host plant. To take account of these factors, we considered a fixed population of *H* plants comprising healthy (*S*(*t*)) and infected (*I*(*t*)) individual plants (i.e. *S*(*t*) = *H* − *I*(*t*)). We formulated the following equations for *V*_*S*_ and *V*_*I*_ (vector density on the average healthy and infected plant, respectively):

2.1 $$\begin{array}{rl} S\;\text{plant colony}\;\frac{\text{d}V_{S}}{\text{d}t} & =\overset{\mathrm{Reproduction}}{\overbrace{aV_{S}\left(1-\frac{V_{S}}{\kappa }\right)}}-\overset{\mathrm{Death}}{\overbrace{bV_{S}}}-\overset{\mathrm{Dispersal}\;\mathrm{loss}}{\overbrace{\theta V_{S}}}\\ & \;+\overset{\mathrm{Dispersal}\;\mathrm{gain}\;(\mathrm{scaled}\;\mathrm{for}\;\mathrm{indiv}.\;\mathrm{plants})}{\overbrace{\theta (V_{S}S+V_{I}I)\frac{S}{S+\epsilon_{3}I}\frac{1}{S}}} \end{array}$$

and

2.2 $$\begin{array}{rl} I\;\text{plant colony}\;\frac{\text{d}V_{I}}{\text{d}t} & =\epsilon_{1}aV_{I}\left(1-\frac{V_{I}}{\epsilon_{2}\kappa }\right)-bV_{I}-\theta V_{I}\\ & \;+\theta (V_{S}S+V_{I}I)\frac{\epsilon_{3}I}{S+\epsilon_{3}I}\frac{1}{I}. \end{array}$$

In equations (2.1) and (2.2) *a* and *κ* denote the low-density net reproduction rate and the maximum vector density per plant for vector multiplication to occur, respectively; *b* denotes the natural mortality rate of vectors; and *θ* denotes the rate of vector dispersal between plants. In addition, *ε*_*j*_ (for *j* ∈ 1, 2, 3) accounts for an increase in the resource quality of infected host plants for vectors if *ε*_*j*_ > 1. This may benefit vector dynamics through an increased vector reproduction rate (*ε*_1_ > 1), increased plant carrying capacity for vectors (*ε*_2_ > 1) or increased vector acceptance of probed plants (termed here as *preference* for infected plants) (*ε*_3_ > 1) (note that for comprehensiveness *ε*_*j*_ < 1, representing decreased plant quality, is also possible in our formulation).

Note that virus modifications may also alter insect preference with respect to feeding retention of infected insects for healthy plants, and of uninfected insects for infected plants. Such traits, which can involve pathogen modification of the insect vector, are not our focus here, and have been discussed elsewhere [17,18]. Nevertheless, for completeness, see electronic supplementary material, appendix S2 for an outline of how this form of modification can be incorporated in our framework and for an explanation of why such traits are not associated with pathogen-mediated insect superabundance.

The pathogen is carried between host plants by insect vectors as they disperse over landscapes. When we consider PMiS, we are referring to elevated insect abundance at the landscape scale that is associated with the incidence of infection among plants. Accordingly, we define the degree of pathogen-mediated insect superabundance, denoted *M*(*I*), in terms of the total population size of the vector in the population of host plants, as the following conditional ratio:

**Degree of PMiS**:

2.3 $$\begin{array}{ll} & M(I)=\frac{V_{S}^{*}(I)S(t)+V_{I}^{*}(I)I(t)}{V_{S}^{*}(0)H}\\ & \;\left\{ \begin{array}{ll} >1\; & \text{pathogen-mediated superabundance}\\ =1\; & \text{no effect of pathogen on abundance}\\ <1\; & \text{pathogen-mediated subabundance.} \end{array}\right. \end{array}$$

The magnitude (degree) of PMiS is high when vector population size in the endemic landscape, i.e. the numerator in equation (2.3), is high compared with its size in the infection-free landscape, i.e. the denominator in equation (2.3). Note that in the above equations we take *V*_*S*_ (and *V*_*I*_) at its dynamic attractor, i.e. $V_{S}^{*}(I)$ (and $V_{I}^{*}(I)$), as the epidemic, *I*(*t*), spreads. This assumption implies that vector density on individual plants reaches a steady state faster than the spread of infection among plants. The assumption has been relaxed in representative simulations to confirm the robustness of the main conclusions.

### 2.2. Epidemiological dynamics

For the majority of insect-borne plant pathogens, the overall transmission rate to plants is proportional to the number of infected vectors that are feeding on individual healthy plants, denoted $V_{S}^{+}$ (figure 1*a*), i.e.

2.4 $$\text{inoculation rate}:\;r^{\mathrm{inoc}}SV_{S}^{+},$$

where *r*^inoc^ is the per infected vector rate at which plants are inoculated during feeding. The total number of infected insects that are feeding on healthy plants ($SV_{S}^{+}$) can be expressed as *Yp*_*S*_, where *Y* is the total number of infected vectors in the local population of host plants and *p*_*S*_ denotes the probability that infected vectors are found on healthy plants. Conversely, the transmission rate to vectors (also referred to as the acquisition rate) is proportional to the number of uninfected vectors that are feeding on individual infected plants, denoted $V_{I}^{-}$ (figure 1*a*), i.e.

2.5 $$\text{acquisition rate}:\;r^{\mathrm{acq}}IV_{I}^{-},$$

where *r*^acq^ denotes the per uninfected vector rate at which the pathogen is acquired during feeding on infected host plants. The total number of uninfected insects feeding on infected plants ($IV_{I}^{-}$) can be expressed as *IV*_*I*_ − *Yp*_*I*_. In this work, we use the expected proportion of the infected insect’s life spent on a healthy plant, denoted *ρ*_*S*_ (or alternatively on an infected plant denoted *ρ*_*I*_), as a proxy for the probability that infected vectors are found on healthy (or infected) plants (i.e. we substitute *p*_*S*_ = *ρ*_*S*_ and *p*_*I*_ = *ρ*_*I*_ in equations (2.4) and (2.5); see electronic supplementary material, appendix S1 for the derivation). Using expected lifespan proportions in this way (e.g. [19]) greatly simplifies calculations without impacting on accuracy (as we have confirmed using complementary computer simulations).

Combining the terms for the inoculation and acquisition rates, and taking account of the expected duration of insect and plant infections, the epidemic is described by equations for the number of pathogen-infected plants and for the number of pathogen-infected vectors at time *t*, i.e. *I*(*t*) and *Y*(*t*),

2.6 $$\mathrm{Pathogen}\text{-infected }\mathrm{plants}\;\frac{\text{d}I}{\text{d}t}=r^{\mathrm{inoc}}Y\rho_{S}-\delta I$$

and

2.7 $$\begin{array}{l} Pathogen\text{-infected}\;\text{vectors}\;\\ \;\frac{\text{d}Y}{\text{d}t}=r^{\text{acq}}\text{(}IV_{I}-Y\rho_{I}\text{)}-\text{(}\sigma +b\text{)}Y. \end{array}$$

In the above equations, epidemics are limited by the rate at which infected plants cease being infectious, denoted *δ*, through mortality or removal by growers (known as roguing). We assume that dead plants are replaced with healthy plants so that the total population of plants remains constant. In addition, the infectious period of the vector is limited by the rate that vectors cease being infectious (the sum of the constant rates that vectors lose the pathogen, *σ*, and natural mortality, *b*). All parameters are listed and defined in table 1.

**Table 1.** Summary of population variables and parameters. The mathematical model tracks changes in plant and vector population variables (i). Vector processes on infected plants are altered by epidemiological mechanisms of pathogen modification (ii) that may underlie pathogen-mediated superabundance. Pathosystems are characterized by vector and pathogen life history parameters (iii).

**(i) population dynamics (plants, vectors)**	**units**
*Y*, density of infected vectors	per field
*V*_*S*_, vector abundance per average healthy plant	per plant
*V*_*I*_, vector abundance per average infected plant	per plant
*V*^+^, abundance of infected vectors	per plant
*V*^−^, abundance of uninfected vectors	per plant
*M*, pathogen-mediated insect superabundance	degree
**(ii) putative modification mechanisms**	
*ε*_1_, modification of reproduction rate	scaling factor
*ε*_2_, modification of carrying capacity	scaling factor
*ε*_3_, modification of vector retention	scaling factor
**(iii) additional parameters**	
*δ*, plant mortality rate	per day
*b*, vector mortality rate	per day
*θ*, vector dispersal rate	per day
*a*, vector reproduction rate (for 0 vector abundance)	per day
*κ*, vector reproduction limit (upper limit on density)	max vectors per plant
*r*^acq^, rate of acquisition of pathogen	per day
*r*^inoc^, rate of inoculation of pathogen	per day

## 3. Results

**Figure 2.**
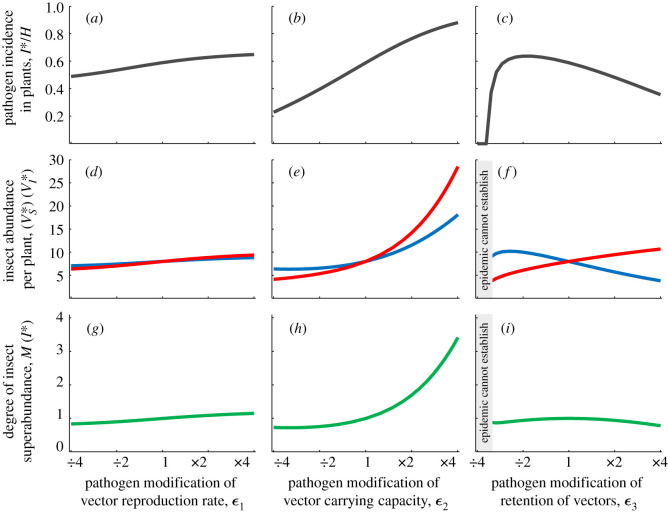
Pathosystem dynamics and insect superabundance: the consequences of pathogen modifications of plant resource quality for vector dynamics and pathogen epidemics (*a*–*i*). When the modifying pathogen is endemic, different levels of modifications (*x*-axis) lead to (*a*–*c*) different values for pathogen incidence among plants; (*d*–*f*) different values of vector abundance per healthy (blue curves) and per infected (red curves) plant; (*g*–*I*) different values for the degree of vector superabundance (green curves). (*a*–*i*) were generated with *K* = 10 over a host plant population size of *H* = 1000; rates per day were *a* = 1, *μ* = 1/5, *r*_acq_ = 1/2, *r*_inoc_ = 1, *δ* = 0.3, *θ* = 2 and *σ* = 2.

We now analyse the effects of the putative epidemiological mechanisms of PMiS to identify those that, when present, are most consistent with PMiS at landscape scales. In figure 2, we show the shapes of response curves relating vector abundance per plant and disease incidence to changes in three critical parameters controlling the epidemiological mechanism of modification. The parameters are pathogen modification of vector reproduction rate (*ε*_1_), of vector carrying capacity (*ε*_2_) and of vector preference for infected plants (*ε*_3_) (see equations (2.1) and (2.2)).

The suppression of plant defences to insects by plant pathogen infection leads to more frequent acceptance of probed plants for sustained phloem feeding. Therefore, defence suppression can effectively increase vector preference for infected plants. We find that, although insect preference for infected plants leads to higher abundances on infected plants than on healthy ones, it lowers the overall incidence of infection among plants. Therefore, increased preference for infected plants leads to a lower overall abundance at the landscape scale when the modifying pathogen is endemic than when no disease is present (i.e. *M* < 1, figure 2*i*). As a corollary, lower preference for infected plants can actually increase incidence as infected vectors encounter healthy plants more frequently (cf. non-monotonic curve in figure 2*c*). At first sight, these results appear counterintuitive, but they are a direct consequence of the effect of insect preference for infected plants. Though it increases the occurrence of pathogen acquisition, it also serves to decrease the overall rate of inoculation to susceptible plants (note the related point that system stability is lost for substantially lower preference because of reduced pathogen acquisition, cf. unstable region, figure 2*c*).

For increased vector carrying capacity of infected plants (*ε*_2_ > 1), however, both the abundance per infected plant (figure 2*e*) and the incidence of pathogen infection among plants (figure 2*b*) are dramatically higher than when infected plants are not modified, leading to vector superabundance (*M* > 1, figure 2*h*). For increased vector reproduction rate on infected plants (*ε*_1_ > 1) a similar pattern to that of increased carrying capacity is found, but the degree of superabundance is very minor (figure 2*g*, cf. figure 2*h*). Therefore, we find that PMiS is most likely to occur for modifications of carrying capacity, and is not expected to arise at all through the modification of insect preference.

What are the implications for testing PMiS in field data? We have shown that PMiS arises through pathogen modification of plant traits that alter insect reproduction, most particularly through the elevation of their insect carrying capacity. A key insight from figure 2 is that when the pathogen modifies such traits then insect abundances per healthy and per infected plant are positively correlated (figure 2*d*,*e* red versus blue curves). The positive correlation occurs for a simple reason: the large insect colonies on infected plants are a source of insects for neighbouring uninfected plants. In other words, local insect dispersal from crowded to less crowded plants tends to reduce insect aggregation on infected plants but increases abundance on neighbouring uninfected plants. As a consequence, it may not be possible to establish statistically significant differences between abundances on healthy and neighbouring infected plants, even when a strongly modifying pathogen leads to a high degree of insect superabundance (e.g. figure 2*h*).

## 4. Discussion

For a number of arthropod-transmitted plant pathogens, infected plants support higher densities of the insect vector than plants that are uninfected in controlled experiments. There is substantial evidence that this synergistic interaction between plants and insect vector is caused by pathogens that modify plant susceptibility to vector colonization [7]. When taken at the scale of fields and landscapes, this interaction may lead to PMiS, but insect superabundance may alternatively be a consequence of environmental factors (i.e. EMiS) or of processes of insect invasion. We developed an epidemiological model to analyse the role of pathogen modification mechanisms in elevated insect vector abundance over landscapes, i.e. ‘superabundance’ [1,2,5,6]. Our modelling showed that only modifications of the vector carrying capacity of infected plants are capable of causing vector superabundance over landscapes. We also found that abundances per healthy and per infected plant are positively correlated in conditions of pathogen-mediated insect superabundance, with implications for the detection of PMiS.

In the case of CMD, which is caused by a *Begomovirus*, an unprecedented increase in the abundance of the whitefly vector, *B. tabaci*, has occurred throughout cassava-growing regions of East and Central Africa since the 1990s [1,5,9]. In some regions, *B. tabaci* abundances on cassava shoot tips changed from a few adults to hundreds [20]. Two principal hypotheses have been advanced to explain this increased abundance, namely: a synergistic interaction between CMD-infected cassava plants and *B. tabaci* [9], or genetic changes in the *B. tabaci* population itself [21]. To date neither has been definitively proven, although the two are not mutually exclusive [2]. Understanding the factors underlying superabundant insect populations, like whitefly in East and Central Africa, is especially important because of the secondary emergence of pathogens (for instance, cassava brown streak virus, which now constitutes a major threat to regional food security) transmitted by shared vector populations [2]. Furthermore, for plant viruses in general, though there is substantial empirical evidence that pathogen infections of plants can increase vector abundance, it is not clear which aspects of the insect life cycle are affected [12–14,22–24].

Using a framework based on the explicit modelling of a general insect vector, through the population dynamics of insect colonies on individual infected and healthy plants, we showed that modifications of vector reproduction but not insect preference can lead to the occurrence of insect superabundance at landscape scales (cf. figure 2). The shapes of the trends in figure 2 demonstrate the simple intuition underlying the result. For PMiS to arise over landscapes not only does abundance per infected plant need to be high, but the incidence of infection among plants also needs to be high. When insects prefer infected plants the abundance per infected plant increases, but the incidence of infected plants decreases (precluding PMiS). When insect reproduction is higher on infected plants, in particular through increased carrying capacity, the abundance per infected plant and the incidence of infected plants are both higher (enabling PMiS). Of the modifications that influence reproduction, increased insect carrying capacity leads to very substantial superabundance while increased per capita reproduction rate leads to only very minor PMiS.

A consequence of the analysis here is that a simplistic approach to detecting evidence for PMiS in which comparisons are made between insect abundance on healthy plants and that on infected plants is prone to error. The reason for this is that colonies on healthy versus infected plants in a field are positively correlated through dispersal (figure 2). In a subsequent paper, we will show how observations of insect abundance over fields on a landscape, together with variation in the incidence of infection among plants in the respective fields, can be used to test more robustly for PMiS. The methods will be applied to field data for a CMD epidemic to shed new light on the original factors underlying *B. tabaci* whitefly superabundance in sub-Saharan Africa.

Although we are motivated by the *Begomovirus*-*B. tabaci* interaction, PMiS may be a more widespread phenomenon among plant pathogens. Accordingly, the simplicity of the framework introduced here, which is based upon pathogen transmission during insect feeding, allows broad qualitative application. An exception to this, however, are the non-persistently transmitted viruses that are acquired during probing by aphids rather than through feeding and hence require a different modelling approach [16]. In addition, numerical predictions for a given insect vector species may also be of interest. For this purpose the framework can be extended to include specific reproductive and behavioural processes for the species of interest, and may incorporate measured effects on insect reproduction and preference (e.g. [14,25]). A further important consequence of PMiS, which is beyond the scope of this work, is its effect on the host range of insect vectors. For instance, broad host ranges are known to occur in all phytophagous insect orders, and, in particular, *B. tabaci*, the vector of cassava mosaic virus, has a relatively wide host range. In future work, which expands upon the present framework, the expected loss or gain in host breadth that constitutes the evolutionary response of insect vectors to PMiS will be analysed.

### 4.1. Conclusion

A common theme underscores the results on PMiS in this paper: superabundance is a landscape measure and as such must be analysed at the scale of landscapes. Thus, we found that evaluating potential epidemiological mechanisms underlying PMiS required a landscape perspective, i.e. their viability depended on their effects on the incidence of infection among plants in fields and not just their abundance on infected plants. Likewise, appropriate methods for testing field data for PMiS must be based on landscape measures. Suitable methods of this kind will take account of variation in the incidence of infection among plants when analysing abundance—assessing abundance on infected plants in relation to abundance on healthy plants alone is insufficient.

## Supplementary Material

Vector location among host plants

## Supplementary Material

Framework extension to encompass pathogen modification of insects

## Data accessibility

This article has no additional data.

## References

[1] Legg JP, Ogwal S 1998 Changes in the incidence of African cassava mosaic geminivirus and the abundance of its whitefly vector along south-north transects in Uganda. J. Appl. Entomol. 122, 169–178. (10.1111/j.1439-0418.1998.tb01480.x)

[2] Legg JP *et al.* 2014 Spatio-temporal patterns of genetic change amongst populations of cassava Bemisia tabaci whiteflies driving virus pandemics in East and Central Africa. Virus Res. 186, 61–75. (10.1016/j.virusres.2013.11.018) 24291251

[3] Macfadyen S *et al.* 2018 Cassava whitefly, Bemisia tabaci (Gennadius)(Hemiptera: Aleyrodidae) in East African farming landscapes: a review of the factors determining abundance. Bull. Entomol. Res. 108, 565–582. (10.1017/S0007485318000032) 29433589 PMC7672366

[4] Kalyebi A *et al.* 2018 African cassava whitefly, *Bemisia tabaci*, cassava colonization preferences and control implications. PLoS ONE 13, e0204862 (10.1371/journal.pone.0204862) 30300388 PMC6177144

[5] Legg JP *et al.* 2011 Comparing the regional epidemiology of the cassava mosaic and cassava brown streak pandemics in Africa. Virus Res. 159, 161–170. (10.1016/j.virusres.2011.04.018) 21549776

[6] Boni SB, Rugumamu CP, Gerling D, Sagary Nokoe K, Legg JP 2017 Interactions between Cassava mosaic geminiviruses and their vector, Bemisia tabaci (Hemiptera: Aleyrodidae). J. Econ. Entomol. 110, 884–892. (10.1093/jee/tox064) 28431093

[7] Eigenbrode SD, Bosque-Perez NA, Davis TS 2018 Insect-borne plant pathogens and their vectors: ecology, evolution, and complex interactions. Annu. Rev. Entomol. 63, 169–191. (10.1146/annurev-ento-020117-043119) 28968147

[8] Carr JP *et al.* 2018 Viral manipulation of plant stress responses and host interactions with insects. Adv. Virus Res. 102, 177–197. (10.1016/bs.aivir.2018.06.004) 30266173

[9] Colvin J, Omongo CA, Maruthi MN, Otim-Nape GW, Thresh JM 2004 Dual begomovirus infections and high Bemisia tabaci populations drive the spread of a cassava mosaic disease pandemic. Plant Pathol. 53, 577–584. (10.1111/j.0032-0862.2004.01062.x)

[10] Dixon AFG 1985 Aphid ecology. London, UK: Blackie & Son Ltd.

[11] Colvin J, Omongo CA, Govindappa MR, Stevenson PC, Maruthi MN, Gibson G, Seal SE, Muniyappa V 2006 Host-plant viral infection effects on arthropod-vector population growth, development and behavior: management and epidemiological implications. Adv. Virus Res. 67, 419–452. (10.1016/S0065-3527(06)67011-5) 17027686

[12] Zhang T, Luan JB, Qi JF, Huang CJ, Li M, Zhou XP, Liu SS 2012 Begomovirus-whitefly mutualism is achieved through repression of plant defenses by a virus pathogenicity factor. Molec. Ecol. 21, 1294–1304. (10.1111/j.1365-294X.2012.05457.x) 22269032

[13] Luan JB, Yao DM, Zhang T, Walling LL, Yang M, Wang YJ, Liu SS 2013 Suppression of terpenoid synthesis in plants by a virus promotes its mutualism with vectors. Ecol. Lett. 16, 390–398. (10.1111/ele.12055) 23279824

[14] Maluta NK, Garzo E, Moreno A, Lopes JR, Fereres A 2014 Tomato yellow leaf curl virus benefits population growth of the Q biotype of Bemisia tabaci (Gennadius) (Hemiptera: Aleyrodidae). Neotrop. Entomol. 43, 385–392. (10.1007/s13744-014-0223-z) 27193818

[15] Thresh JM, Otim-Nape GW, Jennings DL 1994 Exploiting resistance to African cassava mosaic virus. Asp. Appl. Biol. 39, 51–60.

[16] Donnelly R, Cunniffe NJ, Carr JP, Gilligan CA 2019 Pathogenic modification of plants enhances long-distance dispersal of nonpersistently transmitted viruses to new hosts. Ecology 100, e02725 (10.1002/ecy.2725) 30980528 PMC6619343

[17] Gandon S 2018 Evolution and manipulation of vector host choice. Am. Nat. 192, 23–34. (10.1086/697575) 29897804

[18] Mauck K, Bosque-Pérez NA, Eigenbrode SD, DeMoraes CM, Mescher MC 2012 Transmission mechanisms shape pathogen effects on host-vector interactions: evidence from plant viruses. Funct. Ecol. 26, 1162–1175. (10.1111/j.1365-2435.2012.02026.x)

[19] Donnelly R, White A, Boots M 2015 The epidemiological feedbacks critical to the evolution of host immunity. J. Evol. Biol. 28, 2042–2053. (10.1111/jeb.12719) 26285917

[20] Legg JP, Owor B, Sseruwagi P, Ndunguru J 2006 Cassava mosaic virus disease in East and Central Africa: epidemiology and management of a regional pandemic. Adv. Virus Res. 67, 355–418. (10.1016/S0065-3527(06)67010-3) 17027685

[21] Legg JP, French R, Rogan D, Okao-Okuja G, Brown JK 2002 A distinct Bemisia tabaci (Gennadius) (Hemiptera: Sternorrhyncha: Aleyrodidae) genotype cluster is associated with the epidemic of severe cassava mosaic virus disease in Uganda. Mol. Ecol. 11, 1219–1229. (10.1046/j.1365-294X.2002.01514.x) 12074729

[22] Li R *et al.* 2014 Virulence factors of geminivirus interact with MYC2 to subvert plant resistance and promote vector performance. Plant Cell 26, 4991–5008. (10.1105/tpc.114.133181) 25490915 PMC4311212

[23] Yang JY, Iwasaki M, Machida C, Machida Y, Zhou X, Chua NH 2008 *β*, the pathogenicity factor of TYLCCNV, interacts with AS1 to alter leaf development and suppress selective jasmonic acid responses. Genes Dev. 22, 2564–2577. (10.1101/gad.1682208) 18794352 PMC2546693

[24] Lozano-Duran R, Rosas-Díaz T, Gusmaroli G, Luna AP, Taconnat L, Deng XW, Bejarano ER 2011 Geminiviruses subvert ubiquitination by altering CSN-mediated derubylation of SCF E3 ligase complexes and inhibit jasmonate signaling in Arabidopsis thaliana. Plant Cell 23, 1014–1032. (10.1105/tpc.110.080267) 21441437 PMC3082251

[25] Fang Y *et al.* 2013 Tomato yellow leaf curl virus alters the host preferences of its vector Bemisia tabaci. Sci. Rep. 3, 1–5. (10.1038/srep02876) PMC3791452 24096821

