# Supplementary material for: What is pathogen-mediated insect superabundance?
Source: J R Soc Interface. 2020 Sep 9;17(170):20200229. doi: 10.1098/rsif.2020.0229 (PMC7536056; doi:10.1098/rsif.2020.0229)
Supplement: Vector location among host plants [file rsif20200229supp1.pdf]

In this section we provide derivations of a key aspect of our analysis: the expected proportion of an infected insect's lifespan that is spent on healthy vs infected plants. The expected lifespan proportions are used as proxies for the probability that an infected insect is on a particular plant type and hence as closures for the pathosystem through the transmission terms (main text, Eq.s 2.4-2.5).

Supporting Information 1, Vector location among host plants

The probabilities that an infected insect is on a particular plant type, which appear in the acquisition and inoculation rates, main text Eq. 2.4-2.5, can be approximated by accounting for *vector location*, i.e., the proportion of its lifespan (beginning with infection) that an infected vector is feeding on a healthy or an infected plant, denoted ρ_S and ρ_I with $\vec{\rho}$ the vector of these proportions ordered according to $\{S, I\}$. We assume that $\rho_S + \rho_I = 1$, reflecting an assumption that vector dispersal is fast relative to feeding (i.e., in our model insect vectors are either settled on healthy or infected plants with movement between host plants assumed to occur instantaneously relative to feeding). Vector movement between plants is described by a simple linear equation $\frac{d\vec{\rho}}{dt} = A\vec{\rho}$, where the entries $A_{i,j}$ are the rates that the individual vector moves between state i and state j plus the rate of loss from the system in state i when $i = j$, i.e.,

$$A = \begin{pmatrix} -(\sigma + b + \theta) & \theta \frac{S}{S+\epsilon_3 I} \\ \theta \frac{\epsilon_3 I}{S+\epsilon_3 I} & -(\sigma + b + \theta) \end{pmatrix}$$

Since insect infections occur when the insect is feeding on an infected plant, $\vec{\rho}(0)$ is $[0\ 1]^T$. With this initial condition, the linear equation for vector location can be solved for the expected proportions of the infected insect's life spent on each plant type (this is a consequence of the relation $\int_0^\infty \vec{\rho}(t)dt = A^{-1}\vec{\rho}(0)^T$ for linear Markov-chains),

$$\textbf{Vector location} \qquad \vec{\rho}(t) = \qquad [\rho_S\ \rho_I]^T = \left[\frac{\frac{\theta S(t)}{S(t)+\epsilon_3 I(t)}}{\theta + b + \sigma} \quad \frac{\frac{\theta \epsilon_3 I(t)}{S(t)+\epsilon_3 I(t)} + \sigma + b}{\theta + b + \sigma} \right]^T, \tag{S1.1}$$

where the right hand side of Eq. S1.1 is the vector of expected durations on healthy and infected plants divided by the insect's infectious period. The expressions for ρ_S and ρ_I in Eq. S1.1 appear in Eq.s 2.6-2.7, main text, as replacements for p_S and p_I, respectively (cf. transmission terms in Eq.s 2.4-2.5).
