# Supplementary material for: What is pathogen-mediated insect superabundance?
Source: J R Soc Interface. 2020 Sep 9;17(170):20200229. doi: 10.1098/rsif.2020.0229 (PMC7536056; doi:10.1098/rsif.2020.0229)
Supplement: Framework extension to encompass pathogen modification of insects [file rsif20200229supp2.pdf]

In this section we provide a derivation of a framework extension that encompasses insect preference that arises through pathogen modification of insects as well as pathogen modification of plants. The situation may lead to preference of infected insects for healthy plants and preference of uninfected insects for infected plants. This combination is likely to lead to high incidence of pathogen infected plants relative to the case of no preference.

Supporting Information 2, Framework extension to encompass pathogen modification of insects

In the main text *virus modification of plants* alone alters insect preference with respect to feeding retention of insects for infected plants (c.f. ϵ_3 parameter, equations 2.1-2.2). We are mainly interested in virus modification of plants, since empirical evidence for PMiS has been linked to changes in plants. However, *virus modifications of insects*, may alter the preference with respect to feeding retention of infected insects for healthy plants (often termed 'direct' modification) as well as of uninfected insects for infected plants (often termed 'indirect modification'). In this section we outline how these modes of modification may be simultaneously included in our framework. We end this section by indicating why preferences due to 'indirect' and 'direct' modification acting together do not lead to insect superabundance. The main text is focused on the role of virus modifications to the plant environment in leading to insect superabundance. Therefore this section is dedicated to outlining the relevant extension of our framework, and establishing that the inclusion of direct preference does not alter our results.

In this alternative formulation of preference, infected insects may prefer healthy plants (through parameter ϵ_+) and uninfected insects may prefer infected plants (through parameter ϵ_-). The pathosystem dynamics represented by equations 2.1-2.2 and 2.6-2.7, main text, also

apply in this case. However, where previously insect vectors settled on healthy vs infected plants independent of their own infection status (c.f. equations 2.1-2.2 where they settled dependent on the infection status of the plant), when the pathogen modifies insects as well as plants, settling of insect vectors must be altered to depend on insect infection status, i.e.,

$$\frac{dV_S}{dt} = aV_S(1-\frac{V_S}{\kappa}) - bV_S - \theta V_S + \overbrace{\theta(V_S^+ S + V_I^+ I)\frac{S}{S+\epsilon_+ I}\frac{1}{S}}^{\text{Dispersal gain infected insects}} + \overbrace{\theta(V_S^- S + V_I^- I)\frac{S}{S+\epsilon_- I}\frac{1}{S}}^{\text{Dispersal gain uninfected insects}}, \tag{S2.1}$$

$$\frac{dV_I}{dt} = \epsilon_1 aV_I(1-\frac{V_I}{\epsilon_2\kappa}) - bV_I - \theta V_I + \theta(V_S^+ S + V_I^+ I)\frac{\epsilon_+ I}{S+\epsilon_+ I}\frac{1}{I} + \theta(V_S^- S + V_I^- I)\frac{\epsilon_- I}{S+\epsilon_- I}\frac{1}{I}. \tag{S2.2}$$

where V_S^+ and V_S^- represent infected insect and uninfected insect abundance on healthy plants respectively (analogously for abundance on infected plants). In addition, ϵ^+ and ϵ^- represent preference of infected insects for infected plants and preference of uninfected insects for infected plants respectively.

Furthermore, the approximation for the probabilities that an infected insect is on a particular plant type, by accounting for *vector location* (c.f. Eq. $S1.1$), must also be changed to account for modification of insect vectors. Accordingly, the proportion of its lifespan (beginning with infection) that an infected vector is feeding on a healthy or an infected plant, becomes the solution of the simple linear equation $\frac{d\vec{\rho'}}{dt} = A'\vec{\rho'}$, where,

$$A' = \begin{pmatrix} -(\sigma+b+\theta) & \theta\frac{S}{S+\epsilon_+ I} \\ \theta\frac{\epsilon_+ I}{S+\epsilon_+ I} & -(\sigma+b+\theta) \end{pmatrix}$$

Once again, $\vec{\rho'}(0)$ is $[0\ 1]^T$, and the linear equation for vector location can be solved for the expected proportions of the infected insect's life spent on each plant type,

$$\textbf{Vector location} \qquad \vec{\rho'}(t) = \qquad [\rho'_S\ \rho'_I]^T = \begin{bmatrix} \dfrac{\frac{\theta S(t)}{S(t)+\epsilon_+ I(t)}}{\theta + b + \sigma} & \dfrac{\frac{\theta \epsilon_+ I(t)}{S(t)+\epsilon_+ I(t)} + \sigma + b}{\theta + b + \sigma} \end{bmatrix}^T . \qquad \text{(S2.3)}$$

Finally, as per the main text, the total number of infected insects that are feeding on healthy plants (SV_S^+) can be expressed as $Y\rho'_S$, where Y is the total number of infected vectors in the local population of host plants, and ρ'_S is the solution from equation S2.3. Furthermore, the total number of uninfected insects feeding on infected plants (IV_I^-) can be expressed as $IV_I - Y\rho'_I$. Thus, the solutions from equation S2.3 can be combined with the expressions in equations S2.1-S2.2 to close the system.

Vector preference due to indirect and direct virus modifications do not lead to pathogen mediated insect superabundance

When infected insects prefer healthy plants and uninfected insects prefer infected plants, it follows that the incidence of infected plants can become very high. This is because both pathogen acquisition and inoculation occur more frequently in this scenario than in the absence of preference. However, despite the high associated incidence of infected plants, this combination of preference traits does not result in PMiS. The simple explanation is as follows: high incidence of infected plants is in turn associated with a high proportion of vectors that are pathogen infected. Thus, the most numerous preference among vectors is for uninfected plants which have become scarce. As a consequence, the degree of superabundance does not rise above one for this combination of preference traits (note that we have confirmed this using representative simulations based upon the above calculations). Moreover, when the two preference traits are allowed to take any preference value (i.e. infected insects can prefer either infected or uninfected plants; uninfected insects can prefer either infected or uninfected plants) the degree of superabundance never rises above one.
